# Investigation of sex-specific effects of apolipoprotein E on severity of EAE and MS

**DOI:** 10.1186/s12974-015-0429-y

**Published:** 2015-12-16

**Authors:** L. Schrewe, C. M. Lill, T. Liu, A. Salmen, L. A. Gerdes, L. Guillot-Noel, D. A. Akkad, P. Blaschke, C. Graetz, S. Hoffjan, A. Kroner, S. Demir, A. Böhme, P. Rieckmann, A. ElAli, N. Hagemann, D. M. Hermann, I. Cournu-Rebeix, F. Zipp, T. Kümpfel, M. Buttmann, U. K. Zettl, B. Fontaine, L. Bertram, R. Gold, A. Chan

**Affiliations:** Department of Neurology, St. Josef-Hospital, Ruhr-University Bochum, Gudrunstr. 56, Bochum, 44791 Germany; Platform for Genome Analytics, Institutes of Neurogenetics & Integrative and Experimental Genomics, University of Lübeck, Lübeck, Germany; Department of Neurology, Focus Program Translational Neuroscience, University Medical Center of the Johannes Gutenberg University Mainz, Mainz, Germany; Department of Vertebrate Genomics, Max Planck Institute for Molecular Genetics, Berlin, Germany; Max Planck Institute for Human Development, Berlin, Germany; Institute for Clinical Neuroimmunology, Medical Campus Grosshadern, Ludwig Maximilian University, Munich, Germany; Inserm U 1127, CNRS UMR 7225, Sorbonne Universités, UPMC Univ Paris 06 UMR S 1127, Institut du Cerveau et de la Moelle épinière, ICM, F-75013 Paris, France; Department of Human Genetics, Ruhr-University Bochum, Bochum, Germany; Department of Neurology, University of Rostock, Rostock, Germany; Centre for Research in Neuroscience, The Research Institute of the McGill University Health Center, 1650 Cedar Avenue, Montreal, QC H3G 1A4 Canada; Department of Neurology, University of Würzburg, Würzburg, Germany; Neuroscience Axis, Research Center of CHU de Québec—CHUL, Department of Psychiatry and Neuroscience, Faculty of Medicine, Laval University, Québec City, QC Canada; Department of Vascular Neurology and Dementia, University of Duisburg-Essen, Essen, Germany; AP-HP, Hôpital de la Pitié Salpêtrière, Département des maladies du système nerveux, F-75013 Paris, France; School of Public Health, Faculty of Medicine, Imperial College of Science, Technology and Medicine, London, UK

**Keywords:** apoE, Gender, Multiple sclerosis, MSSS, Association studies in genetics

## Abstract

**Background:**

Despite pleiotropic immunomodulatory effects of apolipoprotein E (apoE) *in vitro*, its effects on the clinical course of experimental autoimmune encephalomyelitis (EAE) and multiple sclerosis (MS) are still controversial. As sex hormones modify immunomodulatory apoE functions, they may explain contentious findings. This study aimed to investigate sex-specific effects of *apoE* on disease course of EAE and MS.

**Methods:**

MOG_35-55_ induced EAE in female and male *apoE*-deficient mice was assessed clinically and histopathologically. *apoE* expression was investigated by qPCR. The association of the MS severity score (MSSS) and *APOE* rs429358 and rs7412 was assessed across 3237 MS patients using linear regression analyses.

**Results:**

EAE disease course was slightly attenuated in male *apoE*-deficient (*apoE*^*−/−*^) mice compared to wildtype mice (cumulative median score: *apoE*^*−/−*^ = 2 [IQR 0.0–4.5]; wildtype = 4 [IQR 1.0–5.0]; *n* = 10 each group, *p* = 0.0002). In contrast, EAE was more severe in female *apoE*^*−/−*^ mice compared to wildtype mice (cumulative median score: *apoE*^*−/−*^ = 3 [IQR 2.0–4.5]; wildtype = 3 [IQR 0.0–4.0]; *n* = 10, *p* = 0.003). In wildtype animals, *apoE* expression during the chronic EAE phase was increased in both females and males (in comparison to naïve animals; *p* < 0.001). However, in MS, we did not observe a significant association between MSSS and rs429358 or rs7412, neither in the overall analyses nor upon stratification for sex.

**Conclusions:**

*apoE* exerts moderate sex-specific effects on EAE severity. However, the results in the *apoE* knock-out model are not comparable to effects of polymorphic variants in the human *APOE* gene, thus pinpointing the challenge of translating findings from the EAE model to the human disease.

**Electronic supplementary material:**

The online version of this article (doi:10.1186/s12974-015-0429-y) contains supplementary material, which is available to authorized users.

## Background

Apolipoprotein E (apoE) exerts pleiotropic biological functions, including effects on lipoprotein metabolism as well as on the innate and adaptive immune system. Potential mechanisms underlying the immunomodulatory properties of apoE involve enhanced anti-inflammatory macrophage phenotype, decreased activation of NF-kB and STAT1 [1], and downregulation of TH-1 and TH-17 responses via suppression of pro-inflammatory cytokines secreted by macrophages [2]. apoE is expressed in the CNS and is produced by antigen-presenting cells (dendritic cells, macrophages). These observations have led to the investigation of *apoE* in multiple sclerosis (MS) and its animal model experimental autoimmune encephalomyelitis (EAE; reviewed by [3]). In this context, controversial results have been reported for *apoE* in EAE including both beneficial as well as aggravating effects on disease severity and progression in *apoE* knock-out mice [4, 5]. In parallel, two *APOE* polymorphisms, i.e., rs429358 (ε4, Cys130Arg) and rs7412 (ε2, Arg176Cys), which represent established risk variants in Alzheimer’s disease [6], have been assessed extensively for their role in MS. A recent study compiling data on nearly 30,000 subjects showed that these polymorphisms do not influence MS susceptibility [7]. However, their role in disease progression still remains ambiguous, which at least in part pertains to the fact that the majority of studies have assessed rather small, i.e., underpowered datasets (for an overview see e.g., [8]). Divergent findings may also be due to confounders or effect modifiers such as sex, age, or patient subgroups. Along these lines, a comparatively small study testing 221 patients suggested that the association between *APOE* and MS severity was limited to women [9]; however, this has not been described in other studies [8]. Thus, in the current study, we comprehensively assessed the role of apolipoprotein E on disease severity of EAE as well as MS by taking into consideration potential sex-specific effects of *APOE* genotypes.

## Methods

### Mice, experimental autoimmune encephalomyelitis, histopathology, and quantitative real-time PCR analyses

Animal experiments were approved by the North-Rhine-Westphalia authorities for animal experimentation (AZ 84–02.04.2011.A251). Wildtype (wt) C57BL/6 (Harlan, Germany) and *apoE*^*−/−*^ mice (University of Duisburg-Essen, Germany) were backcrossed to generate littermates. Chronic EAE was induced in male and female 9–11-week-old mice using 100 μg myelin-oligodendrocyte glycoprotein peptide (MOG_35-55_) (Charité Berlin, Germany) emulsified in complete Freund’s adjuvant (CFA) containing 100 μg *Mycobacterium tuberculosis* H37RA (Difco Laboratories, Augsburg, Germany) with pertussis toxin injections (PTX, 100 ng intraperitoneally) (LuBio Science, Luzern, Switzerland) on day 0 (d0) and d2 post-immunization (p.i.). Two independent experiments, each including both genotypes and sexes, were performed. As controls, only littermate animals were used. Clinical EAE signs were evaluated daily using a 10-grade score [10] by an experimenter blinded to the genotype. Concentration of plasma neurofilament heavy chain (NfH) was quantified by ELISA as described previously [11, 12]. The amount of NfH for each animal was calculated as the difference between NfH concentration at d26 after immunization and at baseline (i.e., before induction of EAE). For histopathology, mice were perfused during the chronic disease phase of EAE (d26 or d35), and immunohistochemistry was performed on cryosections of lumbar spinal cord tissue for T cells (rat-α-human CD3, 1:100; AbD Serotec, Düsseldorf, Germany) and macrophages (rat-α-mouse Mac3, 1:100; BD-Pharmingen, Heidelberg, Germany) [13]. Demyelination was assessed using FluoroMyelin™ Red Fluorescent myelin stain (1:300) according to manufacturer’s protocol (Life Technologies, Karlsruhe, Germany) with DAPI counterstaining of nuclei (Southern Biotech, Birmingham, USA). Fluorescent images were captured using an inverted fluorescence microscope (BX51, Olympus), and the percentage of demyelinated area was determined by ImageJ [4]. Data are presented as median [interquartile range [IQR], i.e., 25–75. percentile] or mean ± standard error (SEM). To determine differences in clinical course of EAE, Mann-Whitney *U* test was performed for cumulative median scores (from onset of disease (d8) until the end of observation (d35), statistical significance is graphically indicated as **p < 0.01, ***p < 0.001) as well as median score for individual time points (statistical significance is graphically indicated as #*p* < 0.05). Effects of *apoE* genotypes on NfH were analyzed using Mann-Whitney *U* test and on histological parameters (amount of T cells, macrophages, demyelination, axonal density) using Student’s *t* test.

Quantitative real-time PCR (q-rtPCR) for relative *apoE* expression in the spinal cord of male and female wt mice was performed during the chronic disease phase (d35 p.i.). Total RNA was isolated using TRIZOL followed by the RNAeasy Mini Kit (Quiagen, Hilden, Germany) and transcribed to cDNA according to the manufacturer’s protocol (DNAse1 (Invitrogen, Karlsruhe, Germany); anchored Oligo-dt (Thermo Fischer, Schwerte, Germany); dNTPs (Invitrogen); Superscript II (Invitrogen)). Q-rtPCR was performed on an ABI real-time PCR system (Applied Biosystems, Darmstadt, Germany) using PerfeCTa FastMixII master mix (Quanta Bioscience, Gaithersburg, USA) (primer: Mm01307193_g1, Applied Biosystems) normalized to the housekeeping gene *β-actin* (primer: Mm00607939_s1 Actb, Applied Biosystems) using the ∆∆ct method. Differences of sex and EAE on *apoE* expression were calculated using a one-way ANOVA followed by Bonferroni’s multiple comparison test.

Results were calculated using GraphPad Prism 6 (GraphPad Software, USA). In all experiments, a *p* value of <0.05 was defined as statistically significant, *p* values ≥0.05 as non-significant (n.s.).

### Human subjects and genotyping

All samples were collected after informed written consent and appropriate ethical approval at the respective sites. The current study included 2193 MS cases (70.6 % women) from Germany and 1044 patients (71.0 % women) from France, for whom *APOE* genotypes had been generated previously (see [7] for details) and for whom information on the expanded disability status scale (EDSS) [14], disease duration at the timepoint of EDSS assessment, and age at onset (AAO) was available. In case of multiple EDSS measurements, only the most recent one was used. Based on the available data on the EDSS and disease duration, the multiple sclerosis severity score (MSSS) was calculated [15]. See [7], Additional file 1: Table S1 and Additional file 1: Figure S3 for demographic details.

### Power calculation and genetic association analysis

Power estimates for the human association analyses were calculated using the genetic power calculator [16, 17] assuming a minor allele frequency of 0.15 and 0.07 as reported in NCBI’s dbSNP [18], a type I error rate of 0.05, and an additive quantitative trait locus (QTL) model. For both polymorphisms, our study had excellent (>90 %) power to detect additive QTL variance of 0.5 %. Linear regression analyses using an additive and a recessive model were performed in PLINK v1.07 [19, 20] including all subjects and adjusting for center of recruitment, AAO, and sex. Additional analyses were performed after stratification for sex while adjusting for center and AAO. Empirical *p* values were obtained after 10,000 rounds of permutation. All reported *p* values are two-tailed.

## Results

### apoE deficiency ameliorates EAE course in male but not female mice

**Fig. 1:**
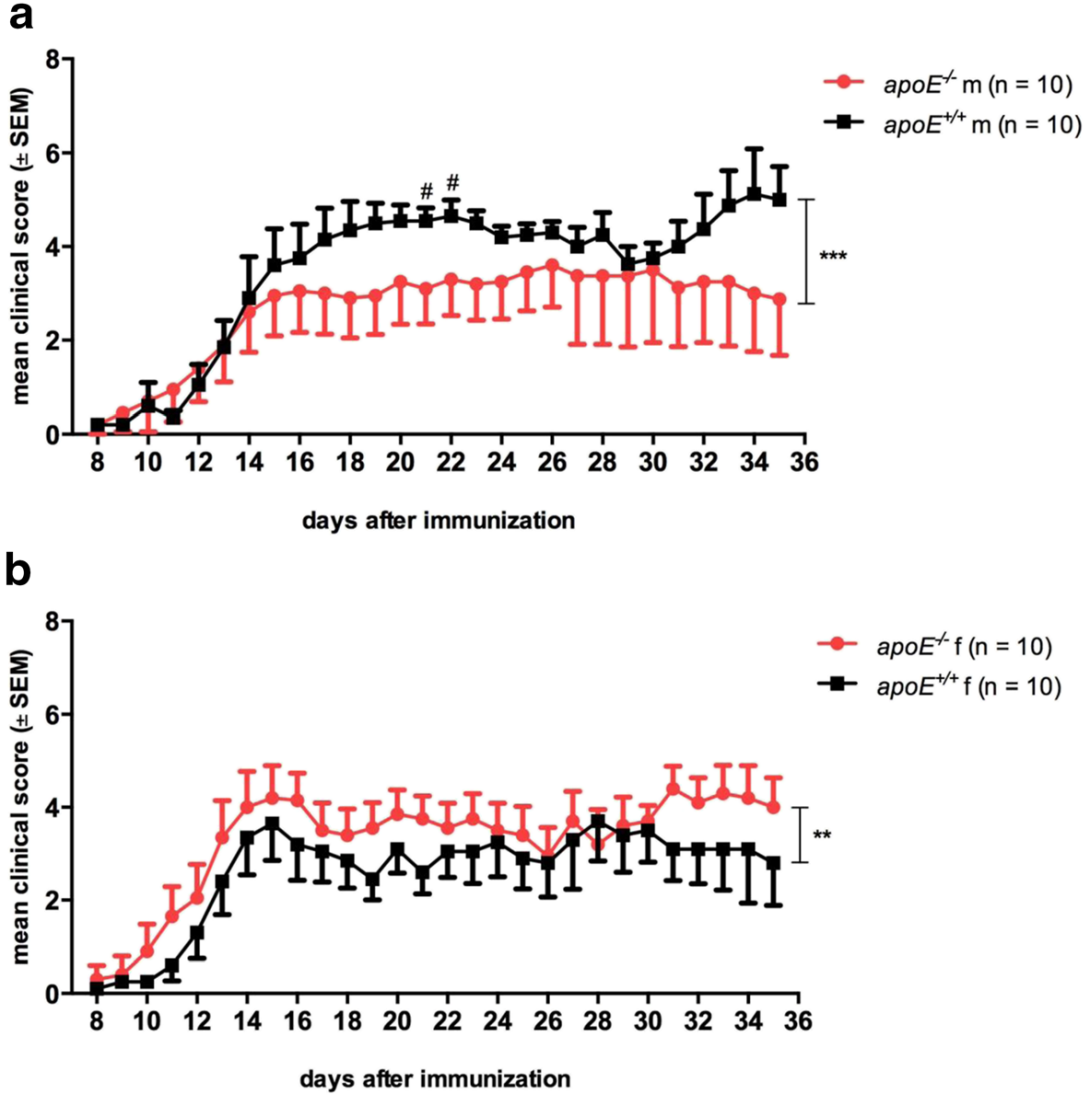
Clinical disease course of active MOG_35-55_ induced EAE in C57Bl/6 wildtype (*apoE* ^*+/+*^) and *apoE*-deficient (*apoE* ^*−/−*^) mice. **a** In male (m) mice, the absence of apoE leads to an attenuated disease severity. **b** In contrast, in females (f), *apoE* deficiency results in a worse disease course. Data were pooled from two independent experiments. Clinical disease course was assessed using a 10-grade scale and depicted as mean ± SEM. Statistical analyses: median scores analyzed with Mann-Whitney *U* test from onset of disease (d8) until the end of observation (d35) (***p < 0.001, **p < 0.01) and for individual time points (#*p* < 0.05)

*apoE* deficiency demonstrated a moderate sex-specific effect on the clinical EAE course. In male animals, cumulative disease severity was attenuated in *apoE*^*−/−*^ mice compared to wt mice (cumulative median score: male *apoE*^*−/−*^ = 2 [IQR 0.0–4.5], *n* = 10, male *apoE*^*+/+*^ = 4 [IQR 1.0–5.0], *n* = 10; *p* = 0.0002; Fig. 1a). Considering median clinical scores for individual time points, male mice showed a significant difference between genotypes on d21 and d22 p.i. (*p* < 0.05). Following this line, body weight was higher in male *apoE*^*−/−*^ mice (cumulative mean weight 28.1 ± 0.9 g) than in male wt animals (cumulative mean weight, 26.2 ± 0.8 g; *p* < 0.001; Additional file 1: Figure S1a). In contrast, in the female group, EAE was more severe in *apoE*^*−/−*^ mice compared to wt controls (cumulative median score: female *apoE*^*−/−*^ = 3 [IQR 2.0–4.5], *n* = 10, female *apoE*^*+/+*^ = 3 [IQR 0.0–4.0], *n* = 10; *p* = 0.003; Fig. 1b and cumulative mean weight: female wt = 20.5 ± 0.7 g; female *apoE*^*−/−*^ = 20.5 ± 0.8 g; *p* = n.s.; Additional file 1: Figure S1b). Differences in EAE incidence supported the sex-specific effect of the *apoE* genotype on disease manifestation (incidence male *apoE*^*−/−*^ = 7/10, male *apoE*^*+/+*^ = 10/10, female *apoE*^*−/−*^ = 10/10, female *apoE*^*+/+*^ = 9/10, *p* = n.s., Fisher’s exact test). These results confirmed our initial observations in pilot studies with non-littermate control animals (data not shown).

**Fig. 2:**
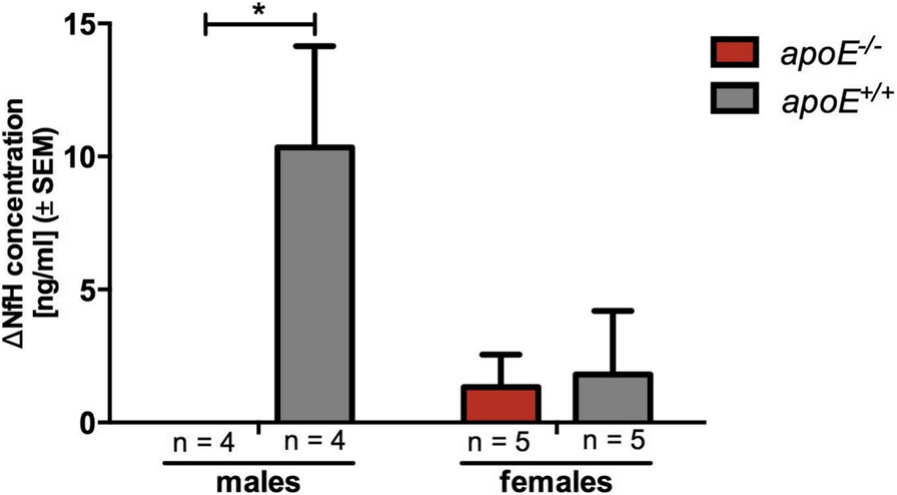
Neurofilament heavy chain (NfH) concentration in blood plasma of C57Bl/6 wildtype mice (*apoE* ^*+/+*^) and *apoE*-deficient (*apoE* ^*−/−*^) mice after a MOG_35-55_-induced EAE indicates the extent of axonal damage. In the chronic phase (d26), male *apoE* ^*+/+*^ mice show a significantly increased NfH concentration compared to male *apoE* ^*−/−*^ mice. NfH concentration was calculated as difference (Δ) between d26 and baseline (i.e., before EAE-induction). Statistical analyses: *p < 0.05 by Mann-Whitney *U* test

In line with the clinical data, neurofilament heavy chain (NfH) concentrations in the plasma, which represent a marker for axonal damage [11, 21, 22] revealed a significant difference between male wt and *apoE*-deficient mice (*p* = 0.0286) but not between female mice (*p* = 0.8; Fig. 2). Significantly increased axonal degeneration in spinal cord sections of male wt animals compared to male *apoE*-deficient mice (d35) was further corroborated by silver impregnation (relative axonal density: male *apoE*^*−/−*^ = 10.2 ± 3.2, *n* = 6, male *apoE*^*+/+*^ = 3.2 ± 0.8, *n* = 5; *p* = 0.001).

Spinal cord immunohistopathology (pooled data from d26 and d35) did not show statistically significant differences of *apoE*^*−/−*^ mice compared to wt mice within the two sex strata; however, observations tended to be consistent with the clinical observations. Specifically, male *apoE*^*−/−*^ mice showed slightly lower macrophage (−29 %, *p* = 0.4, Additional file 1: Figure S2a) and T-cell infiltration (−46 %, *p* = 0.3; Additional file 1: Figure S2b) compared to male wt mice. Likewise, female *apoE*^*−/−*^ mice showed 47 % more infiltrating macrophages (*p* = 0.06; Additional file 1: Figure S2a) and 34 % more T cells (*p* = 0.3; Additional file 1: Figure S2b) than the wt group. Furthermore, male *apoE*^*−/−*^ mice showed a non-significant decrease in demyelination compared to male controls (wt = 8.6 % ± 4.8, *n* = 5, *apoE*^*−/−*^ = 5.3 % ± 5.2; *n* = 5; *p* = 0.3), and a non-significant effect pointing into the opposite direction was observed for females (wt = 2.0 % ± 1.5, *n* = 8, *apoE*^*−/−*^ = 4.3 % ± 3.4; *n* = 5; *p* = 0.1; Additional file 1: Figure S2c).

**Fig. 3:**
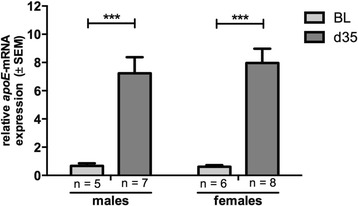
Relative *apoE* expression in the spinal cord at different time points of a MOG_35-55_-induced EAE in C57Bl/6 wildtype mice. Quantitative real-time PCR analyses show an increased *apoE* expression in the chronic phase (d35) of EAE in both females and males compared to untreated animals (BL). Statistical analyses: ***p < 0.001; one-way ANOVA (post test: Bonferroni’s multiple comparison test)

During the chronic EAE phase, *apoE* expression in the spinal cord increased in wt animals in comparison to untreated healthy controls in both strata (females: 13-fold increase; males: 11-fold increase, one-way ANOVA, *p* < 0.001; Fig. 3).

### Association analyses of MSSS and *APOE* polymorphisms do not show statistically significant results

Linear regression analyses of MSSS and *APOE* rs7412 and rs429358 in the overall analyses across 3237 MS patients did not reveal statistically significant (*p* < 0.05) association with MS severity after 10,000 rounds of permutation. This result did not change after stratification for sex (Table 1, Additional file 1: Figure S4). While the minor (C) allele of rs429358 tended to be associated with a higher MSSS across all patients assuming a recessive model (*β* = 0.841, *p*_rec_ = 0.0140) and in the female stratum assuming both recessive (*β* = 0.971, *p*_rec_ = 0.0170) as well as additive models (*β* = 0.221, *p*_add_ = 0.0464), significance of neither of these results survived after 10,000 rounds of permutation (Table 1).

**Table 1:** Association analyses of the MS severity score and *APOE* polymorphisms rs429358 and rs7412

Stratum (N)	SNP	*β*_add_	*p*_add,_ *p*_add-emp_	*β*_rec_	*p*_rec,_ *p*_rec-emp_
All (3237)	rs7412	−0.025	0.824, 0.323	0.010	0.985, 0.453
	rs429358	0.115	0.220, 0.739	0.841	0.0140, 0.774
Women (2290)	rs7412	0.023	0.865, 0.460	0.091	0.883, 0.384
	rs429358	0.221	0.0464, 0.357	0.971	0.0170, 0.765
Men (947)	rs7412	−0.117	0.571, 0.539	−0.009	0.993, 0.923
	rs429358	−0.180	0.302, 0.302	0.446	0.482, 0.998

Linear regression analyses of the MS severity score (MSSS) and *APOE* rs7412 and rs429358 were performed across 3237 MS patients as well as after stratification for sex

*N* number, *SNP* single-nucleotide polymorphism, *add* additive model, *rec* recessive model, *emp p* value obtained after 10,000 rounds of permutation. *β* corresponds to the effect estimated for the minor allele of rs7412 (T) and rs429358 (C), respectively

## Discussion

Results of this study indicate that the absence of apoE slightly attenuates EAE in male mice but at the same time aggravates disease course in female animals. In line with this observation, decreased NfH concentration in male *apoE*-deficient mice in comparison to wt mice suggests an attenuation of axonal damage in male mice lacking apoE. Increased *apoE* expression in the spinal cord of female and male wt mice in the chronic disease phase may indicate an influence of apoE on disease progression during EAE.

In contrast to the results in the rodent model, we did not detect a robust association between MSSS and *APOE* rs7412 or rs429358 in over 3200 patients despite excellent (>90 %) power to observe even moderate changes in the MSSS. This suggests that rs7412 and rs429358 do not have a notable influence on MS severity.

Studies that investigated the role of *apoE* deficiency in EAE have yielded inconsistent, in parts, and even contradictory results [2, 4, 5, 23]. Discrepancies may be due to methodological differences (e.g., the immunization protocol) or due to other modifying factors that have not been investigated in the respective studies. In this context, one potential influencing factor that was not controlled for in previous studies is the sex distribution of the tested animals.

apoE is expressed in the CNS in resident immune cells and has been implicated in different immunoregulatory functions [1, 2, 23]. For instance, in a recent study, milder disease in *apoE*-deficient mice was associated with a reduction of dendritic cells (DCs), which—in turn—can be modulated by sex hormones, i.e., estrogens and primarily E2 [24, 25]. Other studies have reported that apoE modulates macrophages toward an anti-inflammatory phenotype [1] and suppresses microglial activation [26, 27]. The activity of these cells can be modulated by the exposition of estrogen and testosterone (reviewed in [28]). Androgene-receptors (AR) are expressed on immune cells [29]; therefore, especially, androgens may have immunomodulatory or even immunosuppressive effects [28]. A direct interaction of apoE with AR has also been described [30–32]. Thus, immune functions appear to be influenced by androgens via AR and may be modulated by apoE. Although we did not investigate mechanistic pathways, the previously described interactions between immune functions and sex hormones may account for some of the sex-specific differences observed in our study that may additionally be influenced by apoE.

While our human data do not reveal sex-specific association of *APOE* genotypes and MS severity, the association of *APOE* genotypes with Alzheimer’s disease (AD) has been described to be modulated by sex and ethnicity. Whereas *APOE2* and *APOE3* seem to be protective across ethnic groups, *APOE4* increases AD risk [6]. The latter effect appears to be pronounced in women [33].

The lack of association of tested *APOE* polymorphisms with MS severity is in line with the results of most previous publications (for an overview see [8]), including a large pooled re-analysis of previously published datasets on 3518 patients [34] that are independent from those analyzed here. Overall, the authors of the latter study did not find compelling evidence for an association of *APOE* and MSSS either. While they observed a higher MSSS in male homozygote carriers of the *APOE* e4 allele when compared to all other groups (*p* = 0.004), this finding did not withstand multiple comparison corrections [34]. In light of the fact that the two largest, independent, and well-powered studies on rs7412 and rs42935 did not produce robust results, it appears most likely that rs7412 and rs429358 in *APOE* do not play a substantial role in MS severity as measured by the MSSS. However, several aspects need to be considered upon interpretation of our association results: We have tested two *APOE* variants, namely two non-synonymous polymorphisms that represent the most important contributors to Alzheimer’s disease risk [6] and that have been extensively characterized functionally. However, even homozygosity at either of these polymorphic sites does not fully mimic the rodent *apoE* knock-out model [35]. Therefore, the lack of robust genetic effects in humans does not necessarily contradict the results obtained in the *apoE*^*−/−*^ mouse model. However, the translation of findings from experimental models and especially in the context of EAE to the human situation has repeatedly failed as only certain facets of the human disease can be modeled [36]. Thus, we cannot exclude that the effects of apoE observed in the rodent EAE model in this study are of lesser or no relevance for the human disease. In addition, we have only assessed the aforementioned two non-synonymous polymorphisms in the *APOE* region. Thus, we cannot exclude the presence of other variants in the *APOE* locus with a possible effect on MS severity, although a recent genome-wide association study (which did not assess those two variants directly due to technical reasons (see [7] for explanation)), did not observe evidence for an association of MSSS and other genetic variants in the *APOE* region [37]. Another consideration extends to the fact that genetic association analyses of MS severity have overall only yielded rather limited success [38]; one explanation, which could also affect the MSSS association analysis results presented here, is the lack of more appropriate clinical and paraclinical classification schemes to better represent disease severity and progression. In addition, other variables, e.g., information on treatment regimes, may represent confounders in the *APOE* association analysis that could not be accounted for in our and previous analyses (e.g., [34]).

## Conclusions

In conclusion, our study shows a moderate sex-specific influence of *apoE* on EAE severity indicating a complex interaction between *apoE*, sex, and inflammatory processes at least in the animal model. We did not observe robust sex-specific effects of *APOE* polymorphisms on MS severity, which may be explained by several factors including difficulties in comparing rodent *apoE*-deficient animals and polymorphic changes in the human *APOE* gene. Further characterization of *apoE* and its potential sex-specific influences on inflammation may lead to novel insights into disease-modifying mechanisms. Yet, our study highlights difficulties of direct translation of experimental findings in mice to the human situation.

## Additional file

Additional file 1: Figure S1. Body weight of C57Bl/6 wildtype (*apoE* ^+/+^) and *apoE*-deficient (*apoE* ^-/-^) mice during active MOG_35-55_ EAE. **Figure S2.** Histopathological assessment after active MOG_35-55_ EAE. **Table S1.** Demographic details of all included patients. **Figure S3.** Distribution of the MS Severity Score in 3,237 German and French patients. **Figure S4.** Distribution of the MS Severity Score by rs7412 and rs429358 genotypes in all patients and after sex stratification. (PDF 484 kb)
