# Supplementary material for: Investigation of sex-specific effects of apolipoprotein E on severity of EAE and MS
Source: J Neuroinflammation. 2015 Dec 16;12:234. doi: 10.1186/s12974-015-0429-y (PMC4681148; doi:10.1186/s12974-015-0429-y)
Supplement: Additional file 1: Figure S1. — Body weight of C57Bl/6 wildtype (apoE +/+) and apoE-deficient (apoE -/-) mice during active MOG35-55 EAE. Figure S2. Histopathological assessment after active MOG35-55 EAE. Table S1. Demographic details of all included patients. Figure S3. Distribution of the MS Severity Score in 3,237 German and French patients. Figure S4. Distribution of the MS Severity Score by rs7412 and rs429358 genotypes in all patients and after sex stratification. (PDF 484 kb) [file 12974_2015_429_MOESM1_ESM.pdf]

Supplementary Figure 1: Body weight of C57Bl/6 wildtype (*apoE*$^{+/+}$) and *apoE*-deficient (*apoE*$^{-/-}$) mice during active $MOG_{35\text{-}55}$ EAE.

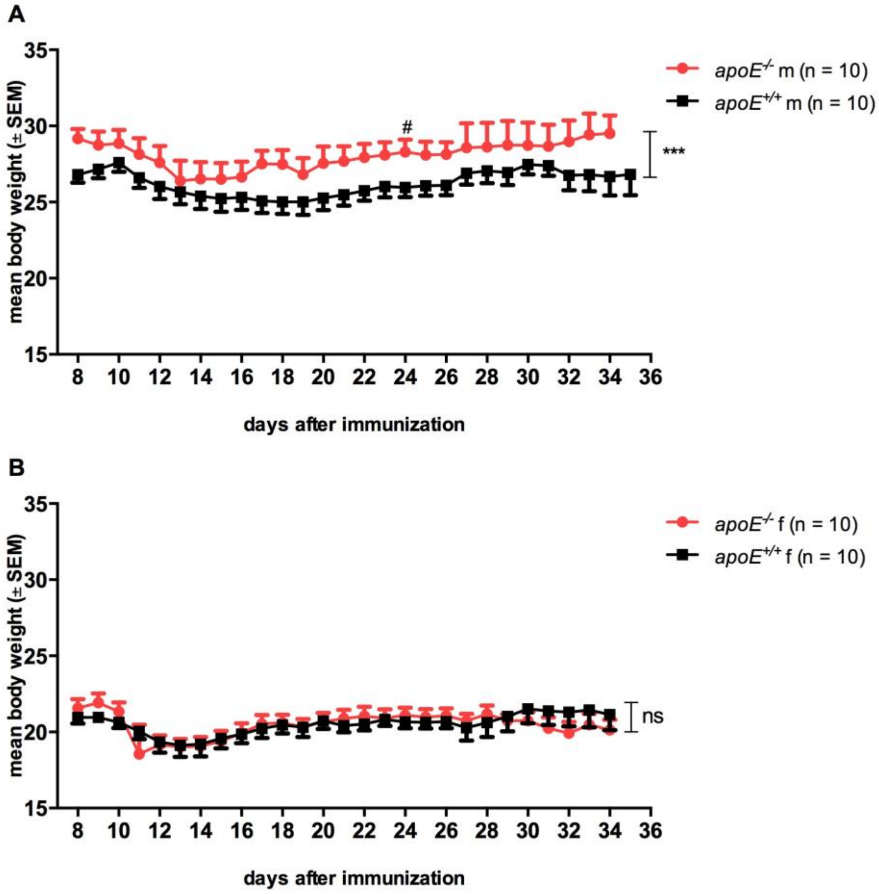

Legend: The graphs show the mean body weight of mice during $MOG_{35\text{-}55}$ induced EAE. (A) Body weight of male (m) *apoE*$^{-/-}$ mice was higher than of *apoE*$^{+/+}$ mice (cumulative mean weight: male *apoE*$^{+/+}$ = 26.2 ± 0.8 g, n = 10, male *apoE*$^{-/-}$ = 28.1 ± 0.9 g; n = 10; $p < 0.001$); (B) In females, body weight was nearly the same in both genotypes (cumulative mean weight: female *apoE*$^{+/+}$ = 20.5 ± 0.7 g, n = 10, female *apoE*$^{-/-}$ = 20.5 ± 0.8 g; n = 10; p = 0.8).

Supplementary Figure 2: Histopathological assessment after active MOG_{35-55} EAE.

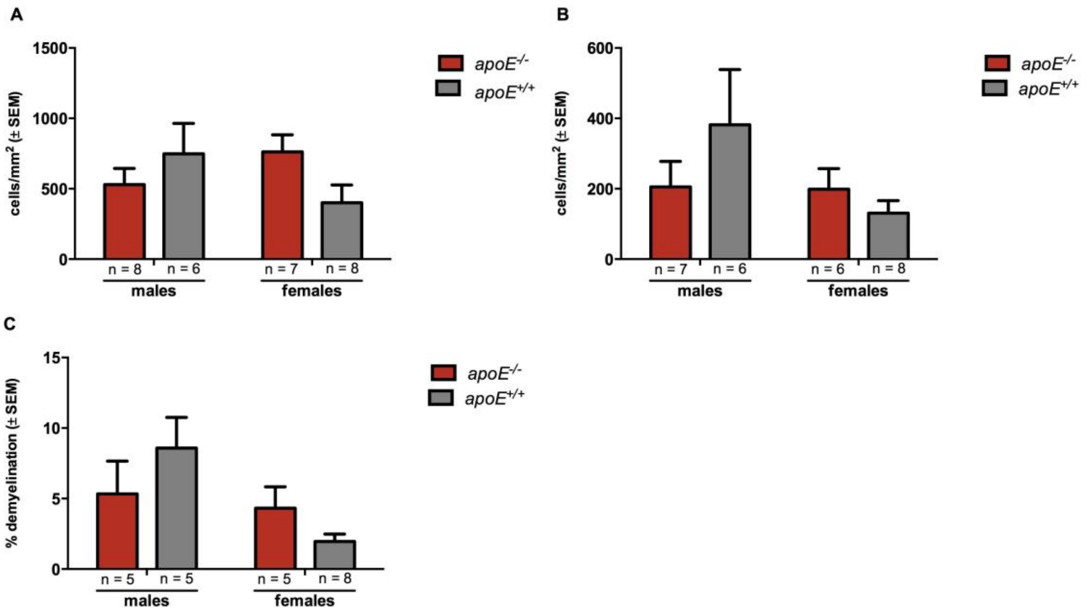

Legend: Quantification of $Mac3^{+}$ cells (macrophages, A); $CD3^{+}$ cells (T cells, B) and demyelinated area (Fluoromyelin™Red, C) in the lumbar spinal cord of female and male wildtype (*apoE*$^{+/+}$) and *apoE*-deficient (*apoE*$^{-/-}$) mice 26 and 35 days after immunization (pooled from both independent EAE-experiments, see manuscript fig. 1A, 1B). Statistical analyses: unpaired t-test yielding no significant differences.

Supplementary Table 1: Demographic details of all included patients.

Center	N cases (% women)	Age at onset (median, IQR)	MSSS (median, IQR)	MAF rs7412 | rs429358
Bochum	688 (70.5)	30 (24-38)	5.15 (2.97-7.33)	0.0932 | 0.1368
Mainz	149 (62.4)	33 (27-41)	6.14 (3.69-7.83)	0.1081 | 0.1443
Munich	409 (73.3)	28 (22-36)	4.35 (2.44-6.46)	0.0860 | 0.1127
Paris	1,044 (71.0)	32 (25-39)	4.55 (2.10-6.81)	0.0768 | 0.1272
Rostock	469 (72.3)	31 (24-39)	3.65 (1.77-6.46)	0.0906 | 0.1239
Wuerzburg	478 (69.5)	27 (21-34)	4.93 (2.87-6.94)	0.0895 | 0.1250
**Total**	**3,237 (70.8)**	**30 (24-38)**	**4.81 (2.44-6.94)**	**0.0867 | 0.1274**

Legend: N = number, IQR = interquartile range, MAF = minor allele frequency (corresponding to the T allele of rs7412 and the C allele of rs429358.

Supplementary Figure 3: Distribution of the MS Severity Score in 3,237 German and French patients.

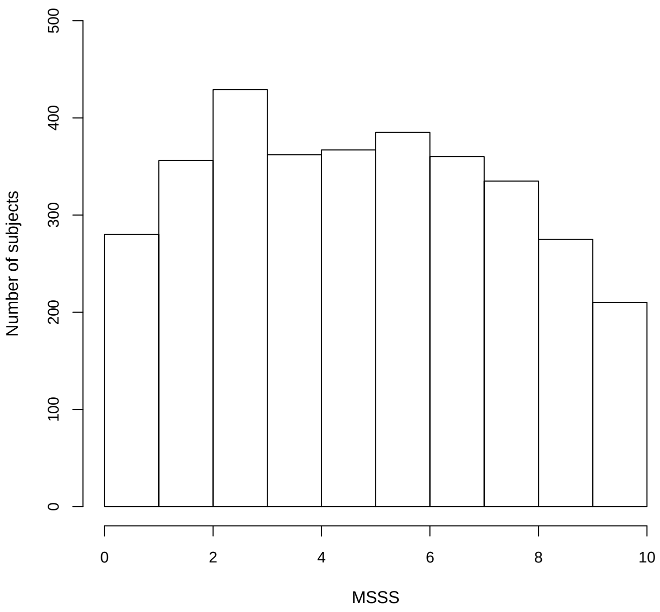

Supplementary Figure 4: Distribution of the MS Severity Score by rs7412 and rs429358 genotypes in all patients and after sex stratification.

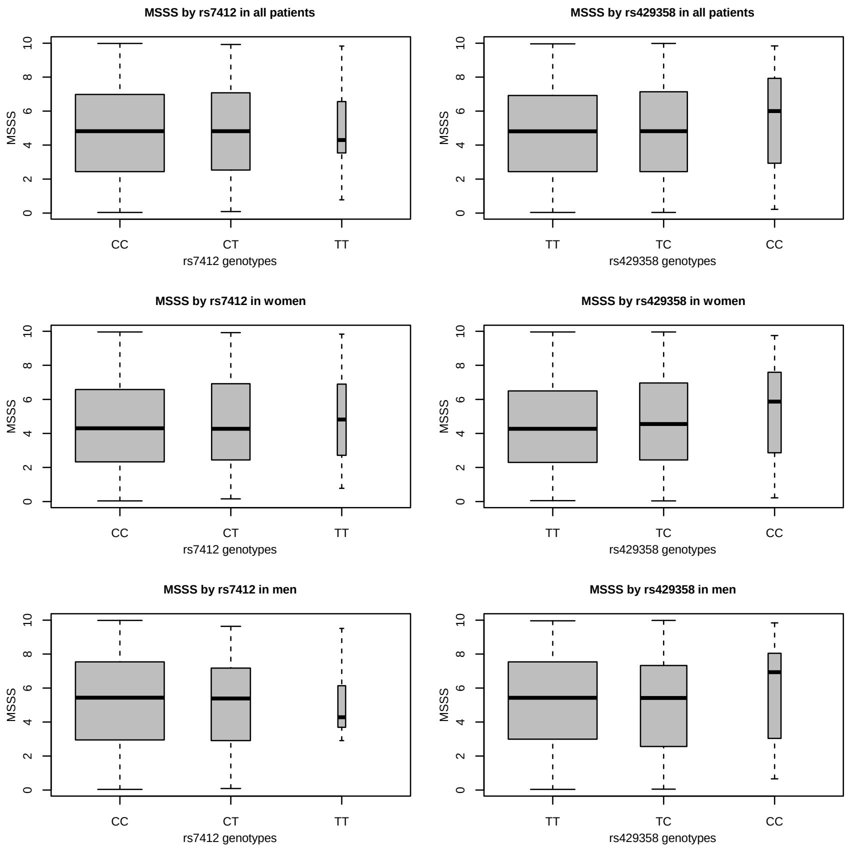

Legend: The box plot shows the distribution of the MS Severity Score (MSSS) dependent on the rs7412 and rs429358 genotypes. Horizontal lines represent median values, boxes represent interquartile ranges, and whiskers extend to 1.5× the interquartile range. Box sizes are scaled to represent the underlying sample size.
